# Comparison of the STANDARD M10 *C*. *difficile*, Xpert *C*. *difficile*, and BD MAX Cdiff assays as confirmatory tests in a two-step algorithm for diagnosing *Clostridioides difficile* infection

**DOI:** 10.1128/spectrum.01662-24

**Published:** 2024-11-29

**Authors:** Hyunseul Choi, Minhee Kang, Sun Ae Yun, Hui-Jin Yu, Eunsang Suh, Tae Yeul Kim, Hee Jae Huh, Nam Yong Lee

**Affiliations:** 1 Biomedical Engineering Research Center, Smart Healthcare Research Institute, Samsung Medical Center, Seoul, South Korea; 2 Center for Clinical Medicine, Samsung Biomedical Research Institute, Samsung Medical Center, Seoul, South Korea; 3 Department of Laboratory Medicine, Seoul Medical Center, Seoul, South Korea; 4 Department of Laboratory Medicine and Genetics, Samsung Medical Center, Sungkyunkwan University School of Medicine, Seoul, South Korea; Taichung Veterans General Hospital, Taichung, Taiwan, China

**Keywords:** *Clostridioides difficile*, M10, Xpert, BD MAX, two-step algorithm

## Abstract

Current guidelines recommend a two-step algorithm rather than relying solely on a single test for diagnosing *Clostridioides difficile* infection. This algorithm starts with enzyme immunoassay (EIA) for detecting glutamate dehydrogenase (GDH) and toxins A/B, followed by nucleic acid amplification test (NAAT) for GDH-positive but toxin-negative cases. This study compared the performance of three commercial NAATs: the STANDARD M10 *C. difficile*, Xpert *C. difficile*, and BD MAX Cdiff assays, utilized as confirmatory testing of the two-step algorithm. Two hundred archived stool specimens, previously tested GDH-positive but toxin-negative by EIA, were analyzed in parallel with these NAATs and toxigenic culture, which served as the reference standard. Sensitivity, specificity, positive predictive value, and negative predictive value were 89.1%, 92.6%, 94.6%, and 85.2%, respectively, for the M10 assay; 95.8%, 86.4%, 91.2%, and 93.3%, respectively, for the Xpert assay; and 89.8%, 91.4%, 93.8%, and 86.0%, respectively, for the BD MAX assay. The rates of invalid results were 1.0%, 0.5%, and 1.0% for the M10, Xpert, and BD MAX assays, respectively. In conclusion, the M10 assay is a reliable diagnostic tool, performing comparably to the Xpert and BD MAX assays when used as confirmatory testing in the two-step algorithm.

**IMPORTANCE:**

While numerous studies have assessed nucleic acid amplification tests (NAATs) as stand-alone tests for diagnosing *Clostridioides difficile* infection, limited research has compared their performance as confirmatory tests in a two-step algorithm. This study evaluated the performance of three commercial NAATs (M10, Xpert, and BD MAX assays) using 200 archived stool specimens initially tested as glutamate dehydrogenase (GDH)-positive but toxin-negative by GDH/toxin A/B enzyme immunoassay, the first step in the two-step algorithm. All three assays demonstrated high sensitivity (89.1% to 95.8%) and specificity (86.4% to 92.6%), with low rates of invalid results (≤1%). Our findings suggest that the M10 assay performs comparably to the Xpert and BD MAX assays when used as confirmatory testing in the two-step algorithm. Offering similar performance and turnaround time to these widely used assays at a slightly lower cost, the M10 assay serves as a practical alternative in this setting.

## INTRODUCTION

*Clostridioides difficile* infection (CDI) is the leading cause of healthcare-associated diarrhea worldwide, resulting in significant morbidity, mortality, and healthcare costs (1–3). Rapid and accurate diagnosis of CDI is essential for effective patient management and infection control. Although toxigenic culture (TC) and cell cytotoxicity neutralization assay (CCNA) are considered the reference standards for diagnosing CDI, their routine use is limited by technical complexity and long turnaround times (TATs) (4, 5). An alternative approach is using enzyme immunoassays (EIAs) to detect glutamate dehydrogenase (GDH) or toxins A/B. Despite their rapid and straightforward nature, these assays lack sensitivity in the case of toxin EIAs and specificity in the case of GDH EIAs (6–8). Nucleic acid amplification tests (NAATs) provide high sensitivity and rapid TATs; however, they are limited in their ability to differentiate between colonization by toxigenic *C. difficile* and true CDI, which could potentially lead to overdiagnosis of CDI (7, 9, 10). The European Society of Clinical Microbiology and Infectious Diseases (ESCMID) guidelines recommend a two-step algorithm rather than relying solely on a single test for diagnosing CDI. This algorithm begins with EIA for detecting GDH and toxins A/B, followed by confirmatory testing using NAAT or TC for cases that are GDH-positive but toxin-negative (4).

The STANDARD M10 *C. difficile* (M10; SD Biosensor, Suwon, Republic of Korea) is a new, fully automated real-time PCR assay that detects the toxin B gene (*tcdB*) in stool specimens. While this assay has recently received Conformité Européenne marking, there are still limited data on its performance. This study aimed to evaluate the performance of the M10 assay as confirmatory testing in the two-step algorithm and to compare it with the widely used, Food and Drug Administration-cleared, fully automated real-time PCR assays: Xpert *C. difficile* (Xpert; Cepheid, Sunnyvale, CA, USA) and BD MAX Cdiff (BD MAX; BD, Franklin Lakes, NJ, USA).

## MATERIALS AND METHODS

### Clinical specimens

This study analyzed 200 archived stool specimens with discordant results (GDH-positive but toxin-negative) in routine *C. difficile* testing. These specimens were collected from 183 patients suspected of CDI at Samsung Medical Center, Seoul, Republic of Korea, from May 2022 to September 2023. The testing method employed in routine *C. difficile* testing was C. DIFF QUIK CHEK COMPLETE (QCC; Techlab, Blacksburg, VA, USA), an EIA for the simultaneous detection of GDH and toxins A/B. Specimens that tested GDH-positive but toxin-negative by this assay were de-identified by removing personal identifiers, including names and medical record numbers, and assigned a unique alphanumeric code. These specimens were stored frozen at –70°C until use in this study. Formed stool specimens (types 1–4 on the Bristol stool scale) and stool specimens with insufficient volume (<1 mL) were excluded from the study. On average, 10 to 12 specimens were thawed each day and analyzed in parallel using the M10, Xpert, and BD MAX assays, along with TC serving as the reference standard.

### GDH/toxin A/B enzyme immunoassay

The QCC assay was conducted according to the manufacturer’s instructions. In brief, 25 µL of the stool specimen was added to a tube containing the diluent and conjugate, and the mixture was transferred to the sample well of the device. After incubation at room temperature for 15 min, the reaction window was washed with washing buffer, followed by the addition of substrate. The results were read after 10 min. A vertical blue line in the antigen and/or toxin side of the reaction window indicated a positive result for GDH antigen and/or toxins A/B.

### Nucleic acid amplification tests

All assays were performed following the manufacturers’ instructions. Briefly, for the M10 assay, a swab was placed into the stool specimen and then into a tube containing pretreatment solution. After vortexing the tube for 10 s, the pretreatment tool was inserted into the sample loading hole of the cartridge. Subsequently, 1.4 mL of the solution was transferred to the pretreatment tool, and the entire volume was injected into the cartridge using the plunger. The cartridge was inserted into the STANDARD M10 instrument, where nucleic acid extraction, amplification, and detection processes were conducted. The total running time was approximately 47 min.

For the Xpert assay, a swab was placed into the stool specimen, inserted into a vial containing the Sample Reagent, and vortexed for 10 s. The entire volume of the Sample Reagent was then transferred into the sample chamber of the cartridge. The cartridge was subsequently loaded into the GeneXpert instrument, where nucleic acid extraction, amplification, and detection processes were conducted. The total running time was approximately 45 min.

For the BD MAX assay, the stool specimen was vortexed for 15 s. Then, using a calibrated loop, 10 µL of the homogenized specimen was transferred into a Sample Buffer Tube and vortexed for 60 s. A Unitized Reagent Strip was placed onto a BD MAX rack for each specimen, with one Extraction Tube and one Master Mix Tube snapped into the strip. Finally, the Sample Buffer Tubes were placed on the rack with the Unitized Reagent Strips, and the rack was then loaded into the BD MAX instrument for automated nucleic acid extraction, amplification, and detection. The total running time was approximately 110 min.

NAAT results were interpreted using the corresponding instrument’s software. Specimens that produced invalid results on initial testing were retested using the original specimens. All tests, including retesting, were conducted as a single assay without any replicates.

### Toxigenic culture

Prior to culturing, stool specimens were subjected to alcohol shock treatment, where the specimens were mixed with an equal volume of anhydrous ethanol and incubated at room temperature for 30 min. The alcohol shock-treated stool specimens were then inoculated onto ChromID *C. difficile* agar (bioMérieux, Marcy-l’Etoile, France) and incubated anaerobically at 35°C for 48 h–72 h. Typical gray-to-black colonies, as well as colonies of other colors or colorless colonies grown on the agar, were confirmed using matrix-assisted laser desorption/ionization time-of-flight mass spectrometry (VITEK MS; bioMérieux). All *C. difficile* isolates were tested for the presence of toxin A and toxin B genes (*tcdA* and *tcdB*) by PCR. DNA extraction was carried out using the boiling method. Briefly, four to five colonies were suspended in 100 µL of sterile distilled water, mixed with 100 µL of DNA extraction buffer (Green Cross Medical Science Corp., Yongin, Republic of Korea), and heated at 96°C for 10 min. After centrifugation at 13,000 × *g* , the supernatant was used as a template for PCR. Conventional PCR for toxin genes was performed using the primers described by Lemee et al. (11). Isolates displaying 369 bp and 160 bp bands in PCR were classified as possessing both *tcdA* and *tcdB* (*tcdA*-positive and *tcdB*-positive strains). Conversely, isolates exhibiting 160 bp and 110 bp bands in PCR were classified as lacking *tcdA* but carrying *tcdB* (*tcdA*-negative and *tcdB*-positive strains). Isolates displaying no bands in PCR were classified as lacking both *tcdA* and *tcdB* (non-toxigenic strains).

If specimens did not show growth of *C. difficile* in culture, they were recultured after alcohol shock treatment, as was done for the initial culture. To increase the yield of *C. difficile* isolation, the specimens were also recultured without alcohol shock treatment; specifically, those not subjected to alcohol shock were inoculating them onto ChromID *C. difficile* agar and incubated anaerobically at 35°C for 48 h–72 h. If NAAT-positive specimens grew *C. difficile* in culture but the isolates were classified as non-toxigenic by toxin gene PCR, the isolates were retested by toxin gene PCR using a suspension of other colonies. If the isolates remained non-toxigenic upon retesting, the specimens were recultured both with and without alcohol shock treatment and then retested by toxin gene PCR. These results were confirmed without further retesting.

### Data analysis

The sensitivity, specificity, positive predictive values (PPVs), and negative predictive values (NPVs) of the M10, Xpert, and BD MAX assays were assessed using TC as the reference standard. Cohen’s kappa values were used to assess the agreement between the NAATs. Specimens that persistently yielded invalid results upon retesting were excluded from the sensitivity, specificity, PPV, NPV, and kappa value calculations for the corresponding NAAT. Correlation between *tcdB* cycle threshold (Ct) values from the NAATs was analyzed using simple linear regression, and the Pearson correlation coefficient (*r*) was calculated. Receiver operating characteristic (ROC) curves were used to assess the ability of Ct values to predict TC results, with optimal Ct cutoff values determined using Youden’s index. Sensitivity, specificity, PPVs, and NPVs for these cutoff values were calculated based on TC as the reference standard. An experienced laboratory technician performed all three NAATs, recording the TATs and hands-on times (HOTs) with a stopwatch. Costs per test for the NAATs were obtained from local suppliers. All statistical analyses were performed using Excel (Microsoft, Redmond, WA, USA) and MedCalc Statistical Software version 19.5 (MedCalc Software Ltd., Ostend, Belgium).

## RESULTS

### Clinical performance

Of the 200 specimens included in this study, 174 (87.0%) yielded growth of *C. difficile* in culture. Of these isolates, 55 (31.6%) were non-toxigenic (*tcdA*-negative and *tcdB*-negative), and 119 (68.4%) were toxigenic (*tcdA*-positive and *tcdB*-positive, *n* = 109; *tcdA*-negative and *tcdB*-positive, *n* = 10) (Table 1).

**TABLE 1:** Detailed test results for the 200 specimens included in this study^*a*^

Toxigenic culture			NAAT			No. of specimens
Culture	*tcdA*	*tcdB*	M10	Xpert	BD MAX	
Pos	Pos	Pos	Pos	Pos	Pos	88
			Pos	Pos	Invalid/Pos	1
			Pos	Invalid/Pos	Pos	1
			Pos	Pos	Neg	5
			Pos	Pos	Invalid/Invalid	1^*b*^
			Neg	Pos	Pos	4
			Neg	Pos	Neg	4
			Neg	Neg	Pos	2
			Neg	Neg	Neg	2
			Invalid/Neg	Neg	Neg	1
Pos	Neg	Pos	Pos	Pos	Pos	10
Pos	Neg	Neg	Pos	Pos	Pos	1
			Neg	Pos	Pos	1
			Neg	Neg	Pos	1
			Neg	Neg	Neg	51
			Invalid/Neg	Neg	Neg	1
Neg	NA	NA	Pos	Pos	Pos	3
			Pos	Pos	Neg	1
			Pos	Neg	Neg	1
			Neg	Pos	Pos	1
			Neg	Pos	Neg	4
			Neg	Neg	Neg	16

^*a*^ NAAT, nucleic acid amplification test; Pos, positive; Neg, negative; NA, not analyzed.

^*b*^ This specimen was excluded from the sensitivity, specificity, PPV, NPV, and kappa value calculations for the BD MAX assay because it yielded an invalid result upon retesting.

Among the 200 specimens analyzed, two (1.0%), one (0.5%), and two (1.0%) initially yielded invalid results in the M10, Xpert, and BD MAX assays, respectively. After retesting, all specimens with initial invalid results were resolved, except for one specimen that remained invalid in the BD MAX assay. Consequently, this specimen was excluded from the sensitivity, specificity, PPV, NPV, and kappa value calculations for the BD MAX assay (Table 1).

The Xpert assay exhibited the highest sensitivity and NPV of 95.8% (95% CI, 90.5%–98.6%) and 93.3% (95% CI, 85.5%–97.1%), followed by the BD MAX assay with sensitivity of 89.8% (95% CI, 82.9%–94.6%) and NPV of 86.0% (95% CI, 78.2%–91.4%), and the M10 assay with sensitivity of 89.1% (95% CI, 82.0%–94.1%) and NPV of 85.2% (95% CI, 77.5%–90.6%). Regarding specificity and PPVs, the M10 assay showed the highest values with specificity of 92.6% (95% CI, 84.6%–97.2%) and PPV of 94.6% (95% CI, 89.1%–97.5%). The BD MAX assay followed with specificity of 91.4% (95% CI, 83.0%–96.5%) and PPV of 93.8% (95% CI, 88.2%–96.9%), while the Xpert assay exhibited specificity of 86.4% (95% CI, 77.0%–93.0%) and PPV of 91.2% (95% CI, 85.7%–94.7%) (Table 2). The Cohen’s kappa values were 0.85 (95% CI, 0.77–0.92) for the M10 and Xpert assays, 0.84 (95% CI, 0.76–0.91) for the M10 and BD MAX assays, and 0.82 (95% CI, 0.74–0.90) for the Xpert and BD MAX assays.

**TABLE 2:** The clinical performance of the M10, Xpert, and BD MAX assays as confirmatory tests in the two-step algorithm^*a*^

Assay	No. of:				Clinical performance, % (95% CI)			
	TP	TN	FP	FN	Sensitivity	Specificity	PPV	NPV
M10	106	75	6	13	89.1 (82.0–94.1)	92.6 (84.6–97.2)	94.6 (89.1–97.5)	85.2 (77.5–90.6)
Xpert	114	70	11	5	95.8 (90.5–98.6)	86.4 (77.0–93.0)	91.2 (85.7–94.7)	93.3 (85.5–97.1)
BD MAX	106	74	7	12	89.8 (82.9–94.6)	91.4 (83.0–96.5)	93.8 (88.2–96.9)	86.0 (78.2–91.4)

^*a*^ CI, confidence interval; TP, true positive; TN, true negative; FP, false positive; FN, false negative; PPV, positive predictive value; NPV, negative predictive value.

### Analysis of discordant results

Among the 119 TC-positive specimens, 18 (15.1%) showed negative results in at least one NAAT. Of these, three were negative in all three NAATs. Among the 81 TC-negative specimens, 13 (16.0%) showed positive results in at least one NAAT, with seven yielding positive results in at least two NAATs. Notably, of these seven specimens, two grew *C. difficile* in culture, but the isolates were classified as non-toxigenic by toxin gene PCR, despite repeated tests (Table 1). A medical chart review revealed that one of the seven specimens negative by TC but positive by at least two NAATs was obtained from a patient who had recently received treatment with an antimicrobial agent known to be effective against *C. difficile* (metronidazole). The remaining six specimens were collected from patients who had not received such antibiotics.

### Analysis of Ct values from NAATs

**Fig 1:**
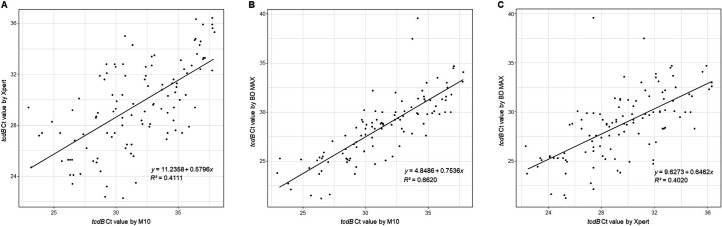
Correlation between Ct values obtained from NAATs. (**A**) Ct values of the M10 assay plotted against those of the Xpert assay (*n* = 111). (**B**) Ct values of the M10 assay plotted against those of the BD MAX assay (*n* = 104). (**C**) Ct values of the Xpert assay plotted against those of the BD MAX assay (*n* = 110).

The associations between Ct values obtained from the NAATs are shown in Fig. 1. Ct values from specimens that were positive for both the M10 and Xpert assays (*n* = 111), both the M10 and BD MAX assays (*n* = 104), and both the Xpert and BD MAX assays (*n* = 110) were included in the analysis. The strongest correlation was observed between the M10 and BD MAX assays (*r* = 0.8136), followed by the M10 and Xpert assays (*r* = 0.6412), and then the Xpert and BD MAX assays (*r* = 0.6340).

**Fig 2:**
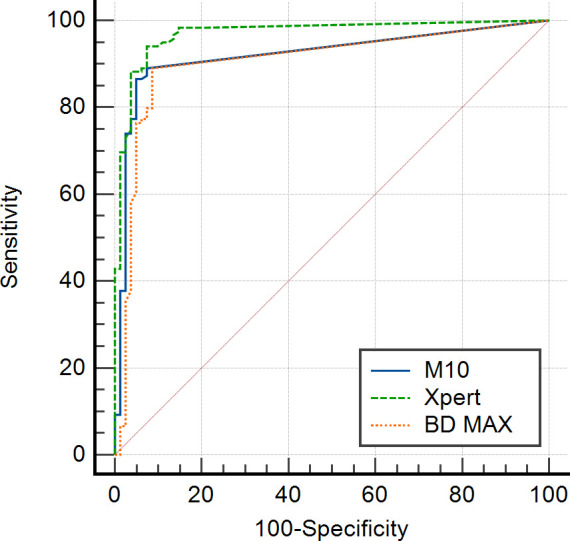
ROC curves assessing the ability of Ct values to predict toxigenic culture results. The blue solid line, green dashed line, and orange dotted line represent the ROC curves for the M10, Xpert, and BD MAX assays, respectively.

The ROC curve analysis revealed areas under the curve (AUC) of 0.921 (95% CI, 0.875–0.955) for the M10 assay, 0.970 (95% CI, 0.936–0.989) for the Xpert assay, and 0.905 (95% CI, 0.856–0.942) for the BD MAX assay (Fig. 2). The optimal Ct cutoff values, calculated using Youden’s index, were 37.9 for the M10 assay, 35.9 for the Xpert assay, and 39.6 for the BD MAX assay. Applying these cutoff values, the M10 assay showed a sensitivity of 89.1% (95% CI, 82.0%–94.1%), specificity of 92.6% (95% CI, 84.6%–97.2%), PPV of 94.6% (95% CI, 89.1%–97.5%), and NPV of 85.2% (95% CI, 77.5%–90.6%). The Xpert assay had a sensitivity of 94.1% (95% CI, 88.3%–97.6%), specificity of 92.6% (95% CI, 84.6%–97.2%), PPV of 94.9% (95% CI, 89.6%–97.6%), and NPV of 91.5% (95% CI, 83.9%–95.7%), while the BD MAX assay exhibited a sensitivity of 89.1% (95% CI, 82.0%–94.1%), specificity of 91.4% (95% CI, 83.0%–96.5%), PPV of 93.8% (95% CI, 88.1%–96.9%), and NPV of 85.1% (95% CI, 77.2%–90.5%) (Table S1).

### Comparison of assay characteristics

Key characteristics for the three NAATs evaluated are summarized in Table 3. HOT and TAT for testing 10 to 12 specimens were shortest for the Xpert assay (24 min and 69 min), followed by the M10 assay (30 min and 77 min), and longest for the BD MAX assay (32 min and 142 min). The cost per test was lowest for the M10 assay (USD 22), followed by the BD MAX assay (USD 24), with the highest cost for the Xpert assay (USD 26).

**TABLE 3:** Key characteristics of the M10, Xpert, and BD MAX assays^*a*^

Characteristic	M10	Xpert	BD MAX
Assay method	Fully automated real-time PCR	Fully automated real-time PCR	Fully automated real-time PCR
Target	*tcdB*	*tcdB*, *cdt*, and *tcdC* Δ117	*tcdB*
Instrument	STANDARD M10	GeneXpert	BD MAX
Throughput	Variable^*b*^	Variable^*b*^	Up to 24 specimens per run
HOT^*c*^	30 min	24 min	32 min
TAT^*c*^	77 min	69 min	142 min
Cost per test^*d*^	USD 22	USD 26	USD 24

^*a*^ HOT, hands-on time; TAT, turnaround time; USD, US dollars.

^*b*^ For the M10 and Xpert assays, the maximum number of specimens that can be processed per run depends on the number of modules available in the instrument.

^*c*^ The HOTs and TATs required for a single technician to test 10 to 12 specimens were evaluated.

^*d*^ Cost per test for each NAAT may vary based on factors such as ordering volume, contracts with suppliers, and other considerations.

## DISCUSSION

Numerous studies have assessed the performance of NAATs as stand-alone tests and/or evaluated the performance of the two-step algorithm utilizing NAATs as confirmatory testing (12–22). However, these studies often included only a limited number of GDH-positive but toxin-negative cases, leading to insufficient data on the performance of NAATs for this specific group, which represents potential candidates for confirmatory testing in the two-step algorithm. Addressing this data gap, this study evaluated the performance of three commercial NAATs (M10, Xpert, and BD MAX assays) on 200 archived stool specimens that tested GDH-positive but toxin-negative by the QCC assay, widely used as the first step in the two-step algorithm. All three assays demonstrated high sensitivity (89.1% to 95.8%) and specificity (86.4% to 92.6%), with low rates of invalid results (≤1%). Kappa values between these assays ranged from 0.82 to 0.85, indicating almost perfect agreement. Our findings suggest that the M10 assay performs similarly to the Xpert and BD MAX assays when used as confirmatory testing in the two-step algorithm.

This study has shown that NAATs can accurately detect toxigenic *C. difficile* missed by toxin A/B EIAs. Among the 200 GDH-positive and toxin-negative specimens analyzed, 119 (59.5%) were confirmed to contain toxigenic *C. difficile* by TC. All three NAATs (M10, Xpert, and BD MAX assays) detected toxigenic *C. difficile* in the majority of these specimens, with sensitivities of 89.1%, 95.8%, and 89.8%, respectively. Due to their high sensitivity, negative NAAT results can effectively rule out the possibility of CDI. The rates of invalid results for the M10 and Xpert assays were 1.0% and 0.5%, respectively, which is comparable to rates reported in previous studies (13, 18, 22). Of note, the rate of invalid results for the BD MAX assay was 1%, considerably lower than rates reported in previous studies (ranging from 4.3% to 5.8%) (13, 15, 18). Overall, the M10 assay is an effective diagnostic tool suitable for confirmatory testing in the two-step algorithm, given its high sensitivity and low rate of invalid results. However, NAATs are unable to distinguish between colonization by toxigenic *C. difficile* and true cases of CDI; thus, clinical evaluation is needed to confirm CDI diagnosis in NAAT-positive/toxin-negative patients (4, 23). Furthermore, conflicting evidence exists regarding the necessity of antimicrobial therapy for NAAT-positive/toxin-negative CDI patients (24–26). Therefore, further studies are warranted to fully assess the clinical utility of NAATs as confirmatory testing in the two-step algorithm.

One approach to addressing the limitations of NAATs, which cannot distinguish between colonization by toxigenic *C. difficile* and true cases of CDI, is to utilize Ct values from these tests. Studies have shown that lower Ct values correlate with the presence of free toxins and greater severity of CDI (27–32). Consequently, the ESCMID guidelines suggest that a CDI diagnosis can be made when a patient exhibits symptoms consistent with the infection and has a positive NAAT result, ideally with a low Ct value (33). However, because our study included only GDH-positive but toxin-negative specimens by EIA, we were unable to perform ROC analysis to assess the predictive ability of Ct values for toxin EIA results. Instead, ROC analysis assessing the predictive ability of Ct values for TC results was conducted, revealing AUCs of >0.9 for all the M10, Xpert, and BD MAX assays. These results suggest that Ct values can effectively predict TC results for all three NAATs.

Currently, numerous commercial NAATs are available for diagnosing CDI, each varying in performance and characteristics such as assay method, cost, HOT, TAT, and throughput (15, 18, 34–36). The M10, Xpert, and BD MAX assays evaluated in this study are fully automated, sample-to-result real-time PCR assays designed to minimize manual steps and improve ease of use. Short HOTs of these assays (24–32 min) can help lower labor costs in the laboratory and provide faster results to clinicians. However, the higher costs of NAATs compared to EIA, TC, and CCNA significantly increase the overall expenses of diagnosing CDI. This impact is evident not only when NAATs are used as stand-alone tests but also as part of a two-step algorithm (37–39). Consequently, laboratories need to select NAATs that offer reasonable costs alongside robust performance. Offering equivalent performance, HOT, and TAT to the widely utilized Xpert and BD MAX assays at a slightly lower cost, the M10 assay presents a practical alternative for confirmatory testing in the two-step algorithm.

TC is considered the reference standard for diagnosing CDI (4, 5); however, this method may not always detect toxigenic *C. difficile* that can be detected by NAATs. In this study, seven specimens confirmed as negative by TC were positive by at least two NAATs. Although classified as false-positive results in this study, these cases likely represent false-negative results of TC because multiple NAATs produced the same positive results. One of these specimens was collected from a patient who had recently received antibiotics known to be effective against *C. difficile*, possibly inhibiting its growth in culture. Interestingly, of these specimens, two grew *C. difficile* in culture, but the isolates were classified as non-toxigenic by toxin gene PCR, despite repeated testing. This discrepancy may be due to the preferential growth of non-toxigenic strains during the culture process.

This study has several limitations. First, we focused solely on assessing the performance of NAATs in cases with discordant EIA results (GDH-positive but toxin-negative). Consequently, additional studies are required to evaluate the performance of NAATs in a wider range of clinical cases, including those that are GDH-positive and toxin-positive. Additionally, we did not determine the strain types or toxin subtypes of *C. difficile* isolates, which may be relevant to clinical outcomes (40–42). Furthermore, we did not use CCNA as the reference standard. While TC is also a recognized reference standard, CCNA has demonstrated a better correlation with clinical outcomes and more accurately defines true cases of CDI (43). Consequently, additional research is needed to assess the performance of the M10, Xpert, and BD MAX assays using CCNA as the reference standard. Another limitation is that the use of archived stool specimens may have influenced both TC and NAAT results. Lastly, this study mainly relied on laboratory data without clinical evaluation to differentiate colonization by toxigenic *C. difficile* from CDI, which is a notable limitation.

In conclusion, our study demonstrates that the M10 assay performs comparably to the widely used Xpert and BD MAX assays when used as confirmatory testing in the two-step algorithm. Offering equivalent performance, HOT, and TAT to these assays at a slightly lower cost, the M10 assay serves as a practical alternative in this setting.

## SUPPLEMENTAL MATERIAL

Table S1 spectrum.01662-24-s0001.docx The performance of the M10, Xpert, and BD MAX assays using optimal Ct cutoff values.

ASM does not own the copyrights to Supplemental Material that may be linked to, or accessed through, an article. The authors have granted ASM a non-exclusive, world-wide license to publish the Supplemental Material files. Please contact the corresponding author directly for reuse.
