# Supplementary material for: Comparison of the STANDARD M10 C. difficile, Xpert C. difficile, and BD MAX Cdiff assays as confirmatory tests in a two-step algorithm for diagnosing Clostridioides difficile infection
Source: Microbiol Spectr. 2024 Nov 29;13(1):e01662-24. doi: 10.1128/spectrum.01662-24 (PMC11705936; doi:10.1128/spectrum.01662-24)
Supplement: Table S1 — The performance of the M10, Xpert, and BD MAX assays using optimal Ct cutoff values. [file spectrum.01662-24-s0001.docx]

Table S1. The performance of the M10, Xpert, and BD MAX assays using optimal Ct cutoff values^a^

| Assay | Optimal Ct cutoff value | Sensitivity,  % (95% CI) | Specificity,  % (95% CI) | PPV,  % (95% CI) | NPV,  % (95% CI) |
| --- | --- | --- | --- | --- | --- |
| M10 | 37.9 | 89.1 (82.0–94.1) | 92.6 (84.6–97.2) | 94.6 (89.1–97.5) | 85.2 (77.5–90.6) |
| Xpert | 35.9 | 94.1 (88.3–97.6) | 92.6 (84.6–97.2) | 94.9 (89.6–97.6) | 91.5 (83.9–95.7) |
| BD MAX | 39.6 | 89.1 (82.0–94.1) | 91.4 (83.0–96.5) | 93.8 (88.1–96.9) | 85.1 (77.2–90.5) |

^a^ PPV, positive predictive value; NPV, negative predictive value; CI, confidence interval.
